# Nutrient-Driven Metabolic Activation and Microbial Restructuring Induced by Endophytic *Bacillus* in Blight-Affected Forest Soils

**DOI:** 10.3390/microorganisms13071454

**Published:** 2025-06-23

**Authors:** Quan Yang, Shimeng Tan, Anqi Niu, Junang Liu, Guoying Zhou

**Affiliations:** 1 Key Laboratory of National Forestry and Grassland Administration on Control of Artificial Forest Diseases and Pests in South China, Central South University of Forestry and Technology, Changsha 410004, China; ywuqun@126.com (Q.Y.); kjc9620@163.com (J.L.); 2 Hunan Provincial Key Laboratory for Control of Forest Diseases and Pests, Central South University of Forestry and Technology, Changsha 410004, China; 3 Key Laboratory of Cultivation and Protection for Non-Wood Forest Trees, Central South University of Forestry and Technology, Changsha 410004, China; 4 State Key Laboratory of Utilization of Woody Oil Resource, Central South University of Forestry and Technology, Changsha 410004, China; 5 College of Forestry, Central South University of Forestry and Technology, Changsha 410004, China; 6 Key Laboratory of Plant Disease and Pest Control of Hainan Province, Institute of Plant Protection (Research Center of Quality Safety and Standards for Agricultural Products of Hainan Academy of Agricultural Sciences), Haikou 571100, China; tshmeng@126.com; 7 College of Life and Environmental Sciences, Central South University of Forestry and Technology, Changsha 410004, China

**Keywords:** endophytic *Bacillus amyloliquefaciens*, *Pinus massoniana* shoot blight, microbial functional diversity, biological control, forest soil health

## Abstract

The climate-driven acceleration of forest disease outbreaks has intensified the demand for sustainable biocontrol strategies. In this study, we evaluated the effects of the endophytic bacterium *Bacillus amyloliquefaciens* csuftcsp75 on soil properties, microbial communities, and functional metabolism in soils affected by *Pinus massoniana* shoot blight. Soil physicochemical analysis, carbon substrate utilization profiling (AWCD), and diversity indices (the Shannon, Simpson, and McIntosh indices) were integrated to assess the microbial responses under different inoculation treatments. The csuftcsp75 treatment significantly improved soil nutrient availability—especially available phosphorus and potassium—and was associated with enhanced microbial metabolic activity and sustained functional diversity. Principal component analysis and correlation mapping revealed strong associations between labile nutrients and microbial responses. Comparative analysis showed that csuftcsp75 promoted a balanced and metabolically rich microbial community, while less compatible strains exhibited transient or unstable effects. These findings support a dual-pathway model wherein nutrient-driven metabolic activation and ecological integration jointly determine biocontrol efficacy. This study highlights the importance of matching microbial inoculants with local soil environments to optimize functional outcomes. This work provides a theoretical basis for applying endophytic *Bacillus* in forest disease management and contributes to the development of ecologically coherent biocontrol strategies.

## 1. Introduction

The spread of plant diseases has intensified with the acceleration of global climate change, with their ecological impact having become increasingly extensive, especially within forest ecosystems. Compared with fruit orchards, where chemical pesticide use is constrained by food safety concerns, coniferous forests—primarily used for timber—face fewer limitations in terms of disease-management options [1]. Although conventional chemical control methods remain widely applied and effective, they are increasingly criticized for their adverse effects on soil ecosystems and plant-associated habitats [2]. Previous studies have shown that chemical pesticides significantly promote fungicide resistance among forest fungi [3]. Another prominent concern is the degradation of soil structure and the health of indigenous microbial communities [4].

Beneficial microbial agents have emerged as promising alternatives in green disease-management strategies and are increasingly employed to control disease outbreaks and enhance forest health [5]. Among these, *Bacillus* spp. has drawn particular attention due to its ability to colonize the rhizosphere and boost plant immunity [6]. In some cases, *Bacillus* strains can also be isolated as endophytes and exhibit strong antagonistic activity against pathogens [7]. These endophytic strains stably colonize tree roots, form biofilms, and function both as antagonists and plant growth promoters [8,9].

*Bacillus amyloliquefaciens* is a well-known soil bacterium with potent antagonistic effects against various plant pathogens [10]. It produces a range of antimicrobial compounds—primarily surfactin, iturin A, bacillaene, macrolactin, etc. [11]—and may reprogram the host plant’s metabolism after colonization to improve growth and stress resilience [12]. Moreover, its application in the rhizosphere may alter the microbial community structure, reduce harmful fungi and, ultimately, improve root zone health and plant growth [13].

Soil microbial community composition and diversity are closely linked to plant growth, nutrient cycling, and immune responses [14]. The introduction of biocontrol strains often leads to shifts in microbial structure and activity, which enhance host resistance to biotic stress [15]. For instance, inoculation with *B. amyloliquefaciens* in maize and buckwheat enhanced nutrient uptake and promoted the accumulation of rare and potentially toxic elements [16]. Similarly, *B. subtilis* inoculation in wheat increased the proportion of beneficial rhizobacteria while suppressing pathogenic fungi [17]. These findings highlight the importance of elucidating how beneficial strains interact with soil properties and local microbial communities to improve the theoretical and practical foundation for biocontrol.

Despite growing interest in microbial biocontrol, the ecological mechanisms driving the functional integration of beneficial bacteria in forest soils remain poorly understood. Few studies have examined how endophytic *Bacillus* strains affect rhizosphere nutrient availability and microbial carbon metabolism in coniferous systems. Their potential to concurrently modulate soil nutrient dynamics, activate microbial metabolism, and enhance disease resilience is still underexplored. This study investigates the ecological functions and biocontrol potential of endophytic *B. amyloliquefaciens* csuftcsp75 in forests affected by dieback disease. Specifically, we assessed: (i) the impact of csuftcsp75 inoculation on key soil nutrients, including total and available forms of nitrogen, phosphorus, potassium, and carbon; (ii) changes in microbial metabolic activity and functional diversity using Biolog EcoPlate profiling; and (iii) correlations between microbial indices and nutrient availability, aiming to clarify how beneficial strains integrate into and reshape rhizosphere function. The findings provide mechanistic insight into *Bacillus*-mediated biocontrol and support sustainable forest disease management.

## 2. Materials and Methods

### 2.1. Assessment of Effects of Csuftcsp75 Strain on Soil Environment

The tested bacterial strains included four endophytic biocontrol agents that are effective against *Pinus massoniana* shoot blight; namely, csuftcsp4, csuftcsp32, csuftcsp59, and csuftcsp75 (GenBank accession number: PQ511142). The pathogen strain used in the experiment was *Diplodia sapinea* (GenBank accession number: PQ511143). All strains were previously isolated and preserved in our laboratory.

*P. massoniana* seedlings were grown in flowerpots measuring 24 cm in diameter and 30 cm in height, with one seedling planted per pot.

The potted soil used in the experiment was collected from a *P. massoniana* plantation near the Xiangtianhua Company, located in You County, Zhuzhou, Hunan Province, China.

Luria–Bertani (LB) and potato dextrose agar (PDA) media were used to cultivate the bacterial and fungal strains, respectively. For revival, the frozen-preserved strains were cultured in shake flasks at 28 °C using the above media, while scale-up cultivation was performed at 25 °C.

The reagents used in the experiment included sodium chloride, 75% ethanol, and 1% sodium hypochlorite. Soil environmental characteristics and microbial diversity were analyzed using Biolog ECO MicroPlates (Supplementary Materials), which were obtained from Biolog Inc. (Hayward, CA, USA).

### 2.2. Evaluation of Effects of Csuftcsp75 Strain on Soil Environment

#### 2.2.1. Pot Experiment

This experiment was conducted at the nursery of the Central South University of Forestry and Technology in Hunan Province, China (112.997752° E, 28.133979° N). The average temperature during the experimental period ranged from 16 °C to 24 °C. The experiment included five treatments, CK, S4, S32, S59, and S75, with each treatment consisting of three pots, and one *Pinus massoniana* seedling planted per pot.

The bacterial strains used (csuftcsp4, csuftcsp32, csuftcsp59, and csuftcsp75) were previously isolated and preserved in our laboratory. The experimental procedure was as follows: the frozen bacterial stocks were first streaked on a PDA solid medium and incubated at 28 °C for over 16 h to obtain single colonies. A single colony was then inoculated into 2 mL of PDB liquid medium in a microcentrifuge tube and cultured at 25 °C and 220 rpm for more than 18 h. This culture was subsequently expanded by inoculating 2% (*v*/*v*) of the bacterial suspension into Erlenmeyer flasks containing fresh PDB medium, followed by incubation at 25 °C and 220 rpm. After expansion, the OD value of the bacterial suspension was adjusted to 0.8 (approximately 10^8^ cfu/mL) using a sterile PDB medium for root drenching.

After transplanting the *P. massoniana* seedlings into pots, 200 mL of the treatment solution was applied per pot. The CK group received an equal volume of sterile PDB as a blank control. The treatment groups were inoculated with culture suspensions of csuftcsp4, csuftcsp32, csuftcsp59, or csuftcsp75, respectively. After inoculation, all plants were watered with clean water every three days.

#### 2.2.2. Determination of Soil Physicochemical Properties

Fourteen days after treatment, each seedling was gently removed from the pot. The bulk soil was shaken off, and the rhizosphere soil was carefully collected using a sterile brush and placed in sterile sealed bags. The samples for soil physicochemical analysis were stored at 4 °C, while the samples for microbial diversity assessment were kept at 20 °C.

Seven parameters were measured to characterize the soil’s physicochemical properties, including total nutrients (total nitrogen, total carbon, total phosphorus, and total potassium) and available nutrients (available nitrogen, available phosphorus, and available potassium). Total nitrogen and total carbon were measured according to the LY/T 1228-2015 standard [18] using an elemental analyzer (EA3100, EuroVector, Pavia, Italy) and a precision balance (Mettler AB235-S, Mettler Toledo, Greifensee, Switzerland). Total phosphorus and total potassium were analyzed using inductively coupled plasma optical emission spectrometry (ICP-OES, Agilent 5800, Santa Clara, CA, USA), following the LY/T 1232-2015 [19] and LY/T 1234-2015 [20] standards, with weighing performed using a Mettler ML204 analytical balance (Mettler Toledo, Switzerland). Available nitrogen was measured using the alkali diffusion method, as described in Bao’s Soil Agrochemical Analysis, and was quantified using a Mettler ML204 balance and a Brand digital titration system. Available phosphorus was determined using the molybdenum–antimony anti-colorimetric method (HJ 704-2014 [21]) with a Multiskan GO 1510 microplate reader (Thermo Fisher Scientific, Waltham, MA, USA). The method for available potassium was the same as that used for total potassium and was also analyzed using ICP-OES.

#### 2.2.3. Soil Microbial Diversity Assessment

The microbial community diversity in the soil samples was evaluated using Biolog ECO MicroPlates. An amount of 1 g of soil was added to a sterile centrifuge tube containing 9 mL of sterile water and shaken at over 500 rpm for 20 min using a benchtop shaker. The suspension was allowed to settle, and the clear supernatant was serially diluted (10^1^, 10^2^, and 10^3^-fold) with sterile water. A 100 µL aliquot of the 10^−3^ dilution was inoculated into each well of the Biolog ECO plate using an eight-channel pipette.

The inoculated ECO plates were incubated at 28 °C for seven days. The absorbance at 590 nm was measured every 24 h using an automated microplate reader. The resulting data were used to calculate the microbial metabolic activity and community diversity indices, including the average well color development (AWCD), Simpson index (D), Shannon index (H), and McIntosh index (U), according to Equations (1)–(4) [22].

(1) $$AWCD=\sum \frac{C_{i}-R_{i}}{n}$$

(2) $$H=-\sum p_{i}(\ln p_{i})$$

(3) $$D=1-\sum {p_{i}}^{2}$$

(4) $$U=\sqrt{\sum {n_{i}}^{2}}$$

In these calculations, *C_i_* represents the absorbance value at 590 nm for each reaction well in the Biolog ECO plate. *R_i_* denotes the absorbance value of the control well in the Biolog ECO plate. Each Biolog ECO plate included three technical replicates, with each replicate containing 31 wells with nutrient substrates and 1 control well; thus, the value of *n* is 31. *P_i_* refers to the proportion of absorbance in the *i*th well (*R_i_*) relative to the total absorbance across all wells in the microplate (ΣRi). *n_i_* indicates the relative absorbance value in the *i*th well, calculated as the difference between Ci and Ri (i.e., *C_i_* − *R_i_*).

### 2.3. Data Processing and Statistical Analysis

All of the measured data were initially organized and standardized using Excel 2016, incorporating data from both the control and treatment groups. An integrated matrix was constructed comprising six soil physicochemical parameters and four microbial metabolic diversity indices (the Shannon, Simpson, McIntosh, and AWCD indices), with three biological replicates per group.

Subsequent data analyses were conducted in R (version 4.4.1) using RStudio Desktop (v2024.04.1+748). The R packages employed included readxl (version 1.4.3), dplyr (version 1.1.4), ggplot2 (version 3.5.1), car (version 3.1−3), vegan (version 2.6−8), and corrplot (version 0.94).

The normality of the variables was assessed using the Shapiro–Wilk test. Variables that did not meet the assumption of a normal distribution were either log-transformed (natural logarithm) or analyzed using non-parametric methods. For comparisons between groups, one-way ANOVA followed by Tukey’s HSD post hoc test was applied to variables meeting both the assumptions of normality and homogeneity of variance (as verified with Levene’s test; *p* > 0.05). For non-normally distributed variables, the Kruskal–Wallis rank-sum test was used in conjunction with Dunn’s multiple comparisons test, with *p*-values adjusted using the Benjamini–Hochberg method.

A correlation analysis between the soil physicochemical properties and microbial diversity indices was performed using Spearman’s rank correlation, and the resulting correlation matrix was visualized via heatmaps, highlighting significant associations (α = 0.05). Redundancy analysis (RDA) was further employed to elucidate the soil–microbe interaction patterns, and the model significance was validated with 999 permutations using the vegan package (version 2.6−8). A biplot was used to represent the spatial relationships between explanatory and response variables. All analyses were based on three independently treated and sampled pots per treatment group. For each pot, one soil sample was used for physicochemical property measurements and microbial metabolic profiling. Biolog ECO plates were used to evaluate the microbial activity, with each plate corresponding to one soil sample. Each carbon substrate on the plate was measured in triplicate wells, and the mean absorbance was used for further analysis.

All statistical procedures incorporated appropriate error control for multiple comparisons and underwent model diagnostics to ensure the robustness and reliability of the results.

### 2.4. Use of Generative AI

ChatGPT (version 4o) and Deepseek (version V3) were used for the purposes of language polishing, R code checking and summarizing the identified literature.

## 3. Results

### 3.1. Effects of Different Treatments on Basic Soil Physicochemical Properties

**Figure 1:**
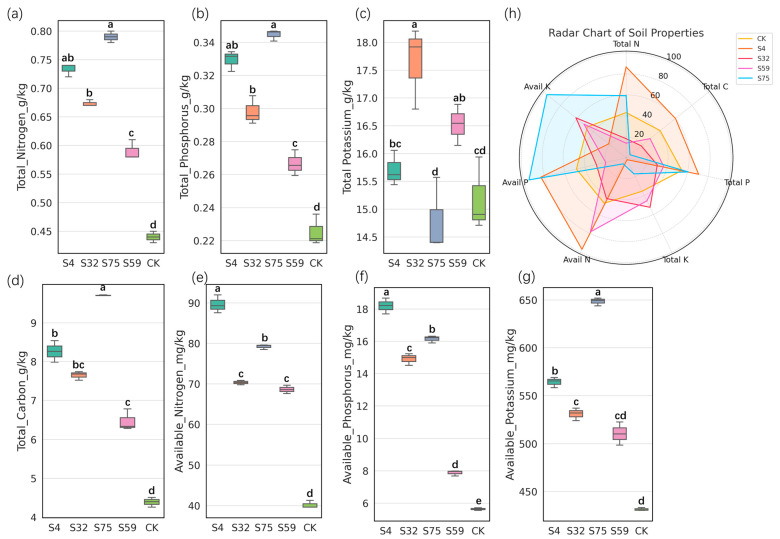
The effects of the different treatments on the soil physicochemical properties. The boxplots illustrate the variations in the (**a**) total nitrogen, (**b**) total phosphorus, (**c**) total potassium, (**d**) total carbon, (**e**) available nitrogen, (**f**) available phosphorus, and (**g**) available potassium contents across the different treatment groups (S4, S32, S75, S59, and CK). Each box represents the interquartile range, the horizontal line indicates the median, and the whiskers represent the data range. A radar chart (**h**) summarizes the relative levels of key soil properties under all treatments. The S75 group exhibited generally higher levels of available nutrients compared with the control (CK), suggesting a potential enhancement of soil fertility under this treatment. The letters above the boxes indicate statistically significant differences between the treatments (*p* < 0.05). Different letters denote significant differences based on the Kruskal–Wallis test, followed by Dunn’s multiple comparisons test. The total phosphorus, available phosphorus, and available potassium parameters were analyzed using ANOVA followed by Tukey’s HSD test.

The results of seven soil parameters, total nitrogen, total carbon, total phosphorus, total potassium, available nitrogen, available phosphorus, and available potassium, across different treatment groups (CK, S4, S32, S59, and S75) are presented in the form of boxplots (Figure 1a–g), with each boxplot representing the comparison of a specific physicochemical property between the treatment groups. In addition, a radar chart (Figure 1h) is used to illustrate the overall effects of each treatment on the soil nutrient status. Here, available nitrogen refers to alkali-hydrolyzable N, available phosphorus refers to Olsen-P (NaHCO_3_-extractable P), and available potassium refers to ammonium acetate-extractable K, all expressed in mg/kg of dry soil. The list of statistical methods used for each parameter is provided in Table 1.

Among most of the measured soil physicochemical parameters, the S75 treatment outperformed the other treatment groups (S4, S32, and S59). As shown in Figure 1a–d, the contents of total nitrogen, total phosphorus, total potassium, and total carbon under the S75 treatment were significantly higher than those in the CK group, which exhibited the lowest levels across all treatments. The total nitrogen and total carbon contents in S75 were also significantly higher than those in S4 and S59, indicating a stronger accumulation of nitrogen and organic matter under this treatment.

Regarding available nutrients (Figure 1e–g), the S75 group showed significantly higher levels of available nitrogen, available phosphorus, and available potassium than all the other treatment groups. Although total potassium did not show a clear advantage, the available potassium content in the S75 group was markedly elevated, suggesting an enhancement in soil potassium exchangeability.

The radar chart (Figure 1h) further highlights the comprehensive effects of the S75 treatment, where the polygonal area covers higher values across all nutrient axes. In contrast, the CK group is confined to the innermost region of the radar plot. Among all treatments, only S75 simultaneously increased both the total and available forms of nitrogen, phosphorus, and potassium, along with a significant elevation in the total carbon content. This indicates that S75 not only improved nutrient availability but also potentially enhanced long-term soil fertility via organic-matter enrichment.

The comparison of the soil physicochemical properties between the treatment groups revealed that root drenching with the candidate bacterial strains significantly affected basic soil characteristics. The S75 treatment group exhibited elevated levels in multiple parameters, which may be attributed to the enhanced nutrient cycling and organic matter decomposition promoted by the introduced bacterial strains [23]. In summary, these results suggest that the S75 treatment exerted a synergistic and comprehensive effect on soil nutrient enrichment, making it the most effective improvement strategy among all the tested treatments.

### 3.2. Analysis of Microbial Metabolic Functions and Temporal Metabolic Dynamics in Soil

The ECO microplate contained a total of 31 different carbon sources and other compounds, which can be categorized into the following groups:

Carbohydrates, such as D-glucose, D-fructose, and D-galactose, serve as primary energy sources that are readily utilized by microorganisms [24].

Amino acids, including L-aspartic acid, L-glutamic acid, and L-phenylalanine, function as nitrogen sources and energy substrates [25].

Organic acids, such as D-malic acid, D-tartaric acid, and D-succinic acid, play important roles in microbial metabolism, being involved in both energy and biosynthetic pathways [26].

Special polymers, including Tween 40 and Tween 60, are more complex compounds that require specific microbial enzymes for degradation and utilization [27].

Phenolic acids, such as salicylic acid and coumaric acid, are less commonly metabolized by general microbes and often require specialized metabolic pathways [28].

Amines, such as putrescine and spermine, act as regulatory molecules in microbial metabolism [29].

The utilization efficiency of each compound was indicated by color changes in the wells during microbial metabolism. For detailed information on the specific compounds and layout of the ECO plate wells, refer to Supplementary Materials Figure S1.

**Figure 2:**
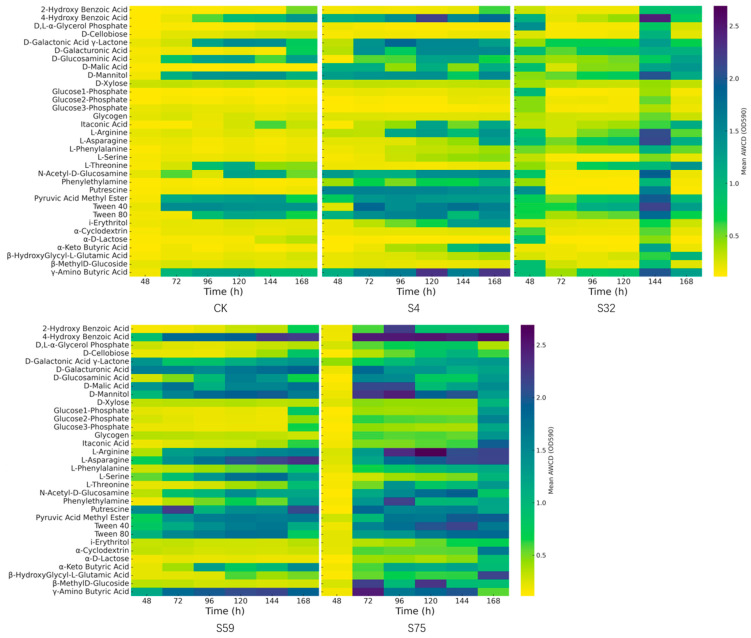
The temporal carbon substrate utilization patterns of the soil microbial communities under the different treatments. The heatmaps display the average color development values (AWCD and OD590) for 31 selected carbon substrate metabolites across the five treatment groups (CK, S4, S32, S59, and S75) over a 168 h incubation period. Each row represents a specific carbon source, including carbohydrates, amino acids, organic acids, polymers, or phenolic compounds. The color intensity indicates the level of substrate utilization, ranging from low (yellow) to high (purple).

We plotted a time-series heatmap of the average color-development rates for all treatment groups (Figure 2). The utilization patterns of 31 metabolic substrates by soil microbial communities under different treatments were visualized using time-series heatmaps, with the metabolic activity indicated by the color-development rates in the microplate wells. These visualizations revealed distinct substrate utilization profiles among the treatment groups. Both the S4 and S75 groups exhibited broader and more intense carbon-source utilization compared with the CK control group.

The microbial communities in the CK group showed limited substrate utilization, with most compounds exhibiting average AWCD (average well color development) values consistently below 1.0 throughout the 168 h period, significantly lower than those of all the other treatment groups (*p* < 0.05). The detectable metabolic activity was mainly restricted to simple carbohydrates (e.g., D-mannitol and glucose-1-phosphate) and a few amino acids, while polymers, organic acids, and phenolic compounds showed almost no detectable color development.

The S4 group demonstrated significantly enhanced metabolic activity across most substrate categories. Strong responses were observed at as early as 48 h and continued to intensify over 168 h. For example, the average AWCD values for γ-aminobutyric acid, β-methyl-D-glucoside, and Tween 80 exceeded 2.0 at 144–168 h, significantly higher than those in the other groups (*p* < 0.05). The greater variability among substrates in S4 also suggested a pronounced increase in microbial functional diversity.

The S75 group exhibited elevated AWCD values across various substrate types, especially carbohydrates and amino acids, with peak reaction rates reaching approximately 1.5–2.0 over 120–168 h. Although S75’s utilization of phenolic compounds was moderate compared with S4, its overall metabolic intensity was significantly higher than that of CK and comparable to S4’s during the later stages (*p* < 0.05). The time-course profile of the S75 group showed a consistent increase from 48 to 120 h, followed by a plateau at 168 h, indicating stabilized metabolic activity.

Furthermore, both the S32 and S59 treatments showed increased AWCD values relative to CK, particularly in carbohydrates and amino acids. However, their responses to complex substrates (e.g., polymers and aromatic acids) were weaker than those observed in S75, and several substrates displayed reduced activity at 168 h compared with earlier time points, suggesting a decline in metabolic intensity over time (*p* < 0.05).

Overall, the qualitative intensity and time-series comparisons in the heatmap support the following conclusions: (1) the S4 treatment significantly enhanced microbial carbon metabolic diversity and activity compared with CK; (2) the S75 treatment produced an even greater enhancement, with notable substrate-specific preferences; and (3) in contrast, the metabolic responses induced by S32 and S59 were transient or limited.

These patterns highlight the distinct influences of the treatments on the soil microbial functional potential, demonstrating a statistically significant substrate utilization specificity and time-dependent variation.

### 3.3. Analysis of Soil Microbial Community Diversity Indices

**Figure 3:**
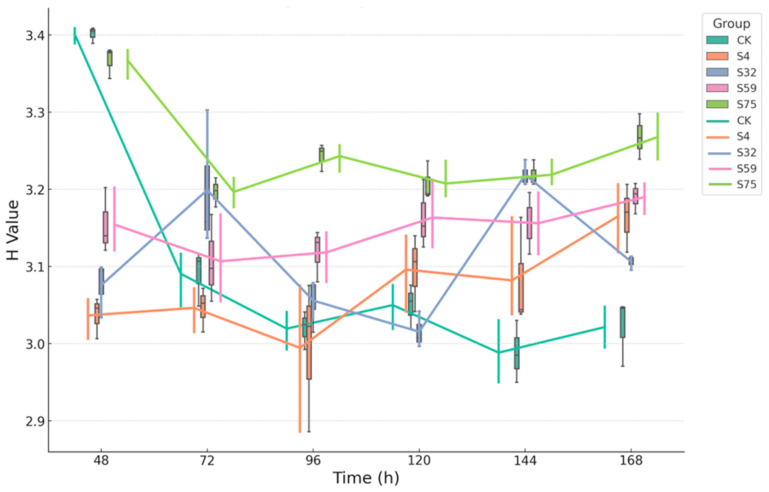
The temporal dynamics of the soil microbial functional diversity (Shannon index) under the different treatments. The boxplot–line charts illustrate the temporal changes in the Shannon index (H-value) of the soil microbial communities under the different treatments (CK, S4, S32, S59, and S75) over a 168 h incubation period. Each line represents the trend of the H-values across the time points, with the endpoints marked by short bars corresponding to the range of the boxplot data, indicating the mean ± standard deviation.

The Shannon diversity index (H-value) revealed distinct temporal differences in soil microbial community diversity across the treatments (Figure 3). The S75 group consistently maintained the highest level of functional diversity, with H-values remaining above 3.2 throughout the entire 168 h incubation, indicating a stable and diverse microbial community. In contrast, the CK group exhibited the lowest H-values, with a sharp decline from 48 to 96 h, followed by a slow recovery, suggesting limited functional richness and adaptability. The S32 and S59 treatments showed moderate and relatively stable diversity over time, while S4 displayed greater fluctuations, initially declining before gradually recovering by 168 h. These patterns indicate that the S75 treatment promoted sustained metabolic activity and functional diversity, whereas the CK group remained functionally constrained, and the other treatments induced moderate or transient effects.

**Figure 4:**
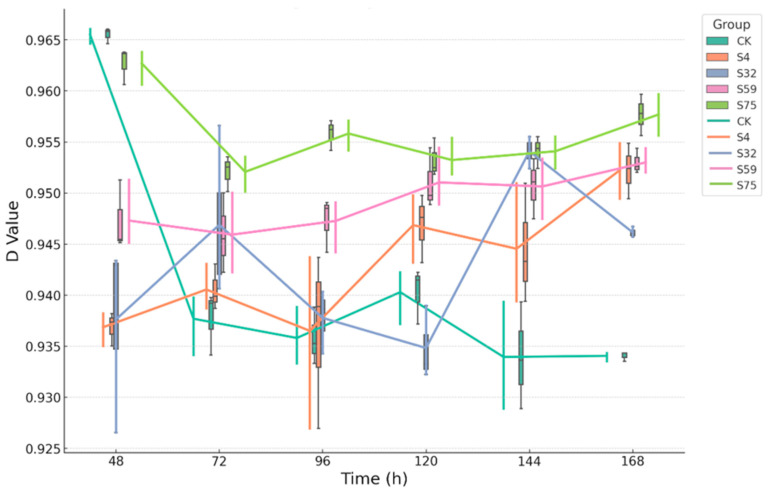
The temporal changes in the Simpson diversity index (D-value) of the soil microbial functional profiles under the different treatments. The combined boxplot–line graphs depict the dynamic changes in the Simpson diversity index (D-value) over a 168 h incubation period across the five treatment groups (CK, S4, S32, S59, and S75). Each point on the line represents the mean ± standard deviation, corresponding to the interquartile range shown in the boxplot at each time point. A higher D-value indicates greater functional evenness and lower dominance within the microbial community.

The Simpson diversity index (D-value) of the soil microbial communities exhibited significant temporal variations between the treatment groups (Figure 4). The S75 group consistently maintained the highest D-values throughout the entire experiment, indicating that root drenching with csuftcsp75 did not disrupt or replace the native microbial community. Instead, it promoted a functionally even and stable community with low dominance. In contrast, the CK group showed a marked decline in its D-value from 48 to 144 h, reflecting increased dominance and reduced functional evenness. The D-values for S32 and S59 remained intermediate between CK and S75, with relatively minor fluctuations and a slight upward trend by 168 h, suggesting a modest recovery of evenness. The S4 group exhibited the greatest variability, with a noticeable decline in the D-value during the middle stage of the experiment (96–120 h), followed by a recovery by the end of the incubation period, reaching levels comparable to those of the S32 group.

**Figure 5:**
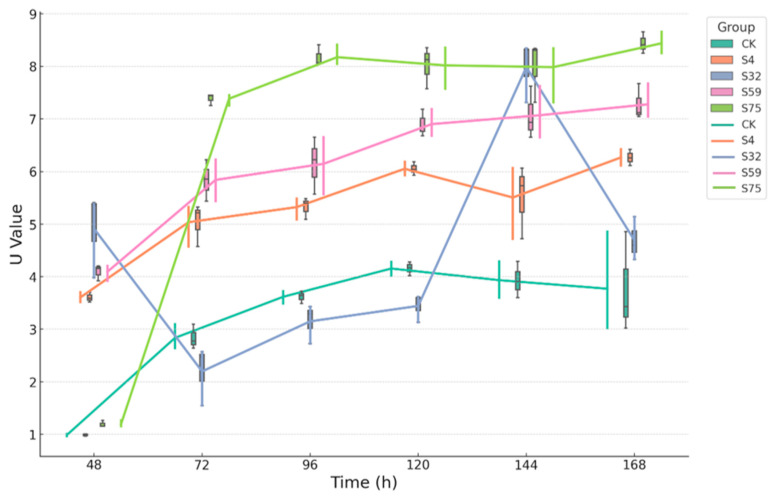
The temporal changes in the McIntosh diversity index (U-value) of the soil microbial functional communities under the different treatments. The combined boxplot–line graphs illustrate the changes in the McIntosh diversity index (U) over a 168 h incubation period across the five treatment groups (CK, S4, S32, S59, and S75). The data are presented as means ± standard deviations. A higher U-value indicates greater overall functional heterogeneity and a more balanced distribution of microbial abundance.

The McIntosh diversity index revealed that the microbial inoculation treatments significantly influenced the temporal dynamics of the soil microbial functional heterogeneity (Figure 5). Among all of the groups, the S75 treatment consistently exhibited the highest U-values with minimal fluctuation, indicating a highly structured and balanced microbial community throughout the experiment. In contrast, the CK group showed the lowest U-values at all time points, with a declining trend between 48 and 144 h. This suggests a loss of microbial evenness and abundance balance, reflecting a reduction in community structural complexity under untreated, natural potted soil conditions. The S32 and S59 groups displayed moderate but stable U-values, while the S4 group exhibited greater temporal variability, with a decline at 96 h followed by partial recovery. These intermediate patterns suggest an improvement in the microbial community structure relative to CK, albeit less pronounced or stable than that observed in the S75 treatment.

A consistent pattern emerged across the Shannon, Simpson, and McIntosh indices: the S75 treatment maintained the highest and most stable levels of soil microbial diversity over time, indicating a functionally rich and well-balanced community. In contrast, the CK group exhibited a continuous decline or low-level fluctuation in diversity, reflecting community instability and reduced metabolic flexibility. The S32 and S59 treatments demonstrated moderate, yet stable, diversity profiles, while the S4 group showed more pronounced fluctuations. Collectively, these patterns suggest that different biocontrol bacterial root-drenching treatments distinctly shape the temporal organization of soil microbial functions in potted soil. Among them, the csuftcsp75 treatment fostered the most resilient and structurally organized microbial community.

#### Correlation Analysis of Soil Physicochemical Properties and Microbial Diversity Among Different Treatment Groups

**Figure 6:**
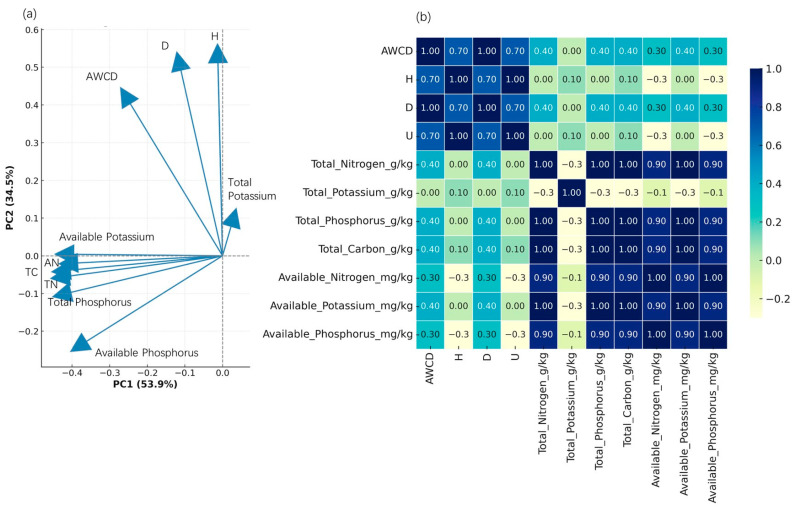
The principal component analysis (PCA) and correlation analysis between soil physicochemical properties and microbial diversity indices. (**a**) The PCA biplot illustrates the relationships between the soil physicochemical variables and the microbial diversity indices under the different treatments (CK, S4, S32, S59, and S75). The arrows indicate the direction and magnitude of each variable’s contribution to the first two principal components (PC1 and PC2), which explain 53.9% and 34.5% of the total variance, respectively. AWCD: average well color development; H: Shannon index; D: Simpson index; AN: available nitrogen; TN: total nitrogen. (**b**) The heatmap of the Pearson correlation coefficients between the microbial diversity indices and the soil chemical properties (total nitrogen, phosphorus, potassium, and carbon). The color intensity represents the strength of the positive or negative correlations, with the corresponding values displayed in each cell. AWCD shows a positive correlation with most soil nutrients, especially the available nutrients. AWCD: average well color development; H: Shannon index; D: Simpson index; U: McIntosh index.

The principal component analysis (PCA) (Figure 6a) results revealed a clear differentiation between the five treatment groups (CK, S4, S32, S59, and S75) based on the soil physicochemical parameters and microbial activity (AWCD). The first two principal components explained 53.9% and 34.5% of the total variance, respectively. The S75 treatment clustered at the positive ends of both PC1 and PC2 and was closely associated with elevated levels of total potassium (TK), available phosphorus (AP), available potassium (AK), and AWCD, suggesting that the microbial activity under this treatment was strongly promoted by improved nutrient availability. In contrast, CK clustered near the center of the PCA plot, associated with lower nutrient values, indicating weak metabolic activation and minimal soil improvement.

In the Pearson correlation heatmap (Figure 6b), AWCD exhibited strong positive correlations with available nitrogen (r = 0.92), available phosphorus (r = 0.94), available potassium (r = 0.97), and total carbon (r = 0.95), implying that labile nutrient pools were key drivers of microbial metabolic activity. Total nutrients such as TN, TP, and TK also showed moderate-to-strong correlations with AWCD (r = 0.67–0.84), reinforcing the conclusion that both total and available forms of soil nutrients jointly shape microbial function.

## 4. Discussion

### 4.1. Soil Nutrients Drive Microbial Functional Activation

Daly et al. [30] integrated multiple studies on mineral, microbial, and metabolic cycles in forest and agricultural soils and proposed a model for the depolymerization and dissolution of organic nitrogen (ON) in soils. Their analysis suggested that microorganisms can utilize polymorphic nitrogen species to facilitate adsorption and desorption on mineral surfaces. Based on the microbial transformation processes of organic nitrogen (including assimilation and mineralization) and the recycling of microbial products, they proposed an integrated plant–microbe–mineral model to regulate soil bioavailable nitrogen. Similarly, in this study, the results from the principal component analysis (PCA) and correlation analysis underscore a comparable ecological mechanism: the metabolic activation of rhizosphere microbial communities is closely tied to the availability and balance of key soil nutrients. Unlike analyses focusing solely on nitrogen variations, we observed from the PCA biplot that variables such as available potassium, available phosphorus, total carbon, and AWCD were strongly and positively loaded in the first two principal components, indicating that these factors also play a major role in explaining the variance among samples. Their co-directional loading suggests a tightly coupled functional axis, where nutrient enrichment is aligned with enhanced microbial activity. Although the biplot itself does not show clustering at the treatment level, the data from the other figures (e.g., Figure 2 and Figure 3) confirm that the S75 treatment resulted in higher levels of these variables. We infer that the soil microbial community under this treatment was more functionally active, occupying a nutrient-rich and functionally active region within the multivariate space [31]. The enhanced nutrient availability observed under the S75 treatment may be partly attributed to the ecological functions of *B. amyloliquefaciens* csuftcsp75. Previous studies have shown that *Bacillus* strains can improve the availability of phosphorous through the secretion of organic acids or phosphatases that solubilize mineral-bound phosphate [32] and they can also enhance potassium mobilization by producing chelating agents or low-molecular-weight organic acids [33]. Additionally, *Bacillus* spp. is known to release metabolites as metabolic activators, stimulating the rhizosphere microbial community and altering root exudation, thereby affecting the overall rhizosphere enzyme environment. These changes in the environment indirectly influence plant-nutrient absorption and environmental adaptability [34].

Correlation analysis further revealed that AWCD was most strongly associated with available potassium (r = 0.97) and available phosphorus (r = 0.94), followed by total carbon and available nitrogen. These results point to bioavailable nutrient pools as proximate drivers of microbial metabolic activation, possibly by fueling substrate assimilation, enzymatic processes, or the growth of metabolically versatile taxa [35]. Such correlations are consistent with the functional roles of *Bacillus* in modifying nutrient bioavailability. The elevated availability of K and P likely reflects both microbial transformation processes and microbe-mediated mineral solubilization. These effects are chemical and biological in origin, emerging from dynamic interactions between introduced strains and native microbial consortia. In contrast, total nutrient pools (e.g., total nitrogen, total phosphorus, etc.), while moderately correlated, appeared to play a more structural rather than functional role. The control group (CK), which exhibited the lowest AWCD and nutrient values in parallel analyses, was characterized by weak microbial activity and limited available nutrients. This observation aligned with the biplot analysis, supporting the idea that the presence of nutrients, alongside their availability and integration into microbial networks, govern functional expression [36].

These findings agree with prior studies showing that readily available phosphorus and potassium are key regulators of microbial community metabolism and diversity, enhancing biomass as well as functional breadth and enzyme activity [37]. Similar patterns have been observed in other studies using *Bacillus amyloliquefaciens* or related species in degraded or nutrient-poor soils. For instance, Chen et al. [38] reported improved available phosphorus and microbial activity in forest soils following *Bacillus* inoculation. In subtropical forest ecosystems, Hu et al. [39] demonstrated that *Bacillus*-treated soils showed enhanced enzyme activity and rhizosphere metabolic potential compared with non-inoculated controls. These parallels suggest that the observed benefits under the S75 treatment are not isolated but align with broader patterns documented in forest microbiome restoration contexts. The results suggest that the enhanced microbial function observed under the inoculated treatments, especially S75, may be attributed to a synergistic mechanism, where improved nutrient accessibility amplified the ecological expression of introduced or stimulated microbial populations.

### 4.2. Integrated Interpretation of Soil Microbial Functional Diversity and Carbon Metabolic Profiles

The integrated assessment of carbon substrate utilization (AWCD) and microbial functional diversity indices (the Shannon H, Simpson D, McIntosh U indices) revealed coherent treatment-specific trajectories, highlighting how inoculation strategies shape both microbial metabolic intensity and structural complexity. It is generally believed that root exudates can significantly alter the structure or function of microbial communities. For example, organic acids and sugars can stimulate rhizosphere microbes’ ability to transform nitrogen [40]. Planting structures and tillage practices may alter the carbon source utilization patterns of soil microbial communities, ultimately systemically affecting the nutritional environment of the entire rhizosphere and further influencing the community structure [41,42]. These patterns closely align with the conceptual model proposed in this study, wherein enhanced carbon metabolism and increased diversity emerged as parallel, interlinked outcomes of rhizosphere restructuring [43].

Among all treatments, S75 consistently supported the highest AWCD values and the most stable diversity indices, indicating a microbial community that was both metabolically active and functionally balanced. The early and sustained activation of carbon metabolism, particularly for labile substrates such as carbohydrates and amino acids (Figure 2), coincided with elevated and stable H-, D-, and U-values across all time points (Figure 3, Figure 4 and Figure 5). This suggests that S75 inoculation stimulated microbial activity and promoted a resilient and ecologically structured community, characterized by high evenness, low dominance, and broad substrate responsiveness.

However, the CK group exhibited minimal carbon substrate utilization, a narrow metabolic profile, and declining diversity over time, indicative of a dormant or compositionally limited microbial community. The decline in the H and U indices from 48 to 96 h, coupled with low AWCD, points to an environment with poor functional recruitment and limited adaptive capacity.

The S32 and S59 treatments followed intermediate trajectories, with moderate substrate utilization and diversity values that remained relatively stable but unremarkable. These results suggest the partial stimulation of microbial function, likely due to moderate shifts in community composition or nutrient conditions, yet insufficient to trigger broader ecological reorganization.

The S4 group displayed a unique biphasic pattern, with early peaks in AWCD and diversity followed by mid-stage declines and partial recovery at later time points. Unlike S75, however, the metabolic and structural instability observed in S4 may not be merely a result of microbial competition, but more likely reflects poor ecological compatibility between the inoculated strain and the native soil environment. The observed fluctuations suggest that the introduced microbe struggled to establish functional integration, potentially due to mismatches in nutrient preferences, root exudate responses, or antagonistic interactions with indigenous taxa. Such a pattern underscores that inoculation success depends on the functional traits of the microbe as well as its capacity to adapt and persist within the local soil–microbe–plant nexus.

These findings reinforce the central premise of our proposed framework: inoculation effects are not limited to the immediate stimulation of microbial activity but extend into the long-term restructuring of microbial diversity and ecological balance. The degree to which these effects manifest (stabilization versus fluctuation; enhancement versus suppression) appears to depend on how effectively the introduced microbial strains integrate into and reshape the local metabolic landscape. While our assessment focused on community-level functional diversity rather than taxonomic composition, the observed stability in the diversity indices (especially Simpson’s D) suggests that enhanced microbial function may have resulted from cooperative integration rather than competitive displacement. This interpretation supports the idea that successful inoculants, such as csuftcsp75, may stimulate existing microbial networks without structurally disrupting indigenous community members.

## 5. Conclusions

This study demonstrated that microbial inoculation (particularly the csuftcsp75 treatment) significantly enhances rhizosphere microbial functional activity, diversity, and structural stability via simultaneous improvements in soil nutrient availability and metabolic responsiveness. Integrating soil physicochemical analyses, carbon substrate utilization profiling, and multidimensional diversity indices, we established that the functional success of inoculation depends on nutrient enrichment as well as the ability of introduced strains to integrate ecologically within the native soil–microbe matrix.

Building on these findings, we proposed a conceptual model in which effective microbial amendments reshape rhizosphere function via a coupled pathway of resource activation and structural reorganization. In this framework, nutrient accessibility—especially in the form of available phosphorus, potassium, and labile carbon—acts as a metabolic catalyst that triggers microbial recruitment and substrate-level activation. When paired with ecologically compatible inoculants, this process fosters a functionally rich and compositionally balanced community capable of sustained metabolic output and adaptive resilience. Conversely, where ecological mismatches occur (as observed in S4), functional instability and diversity fluctuations may arise, highlighting the context-dependent nature of microbial integration.

This study underscores the ecological importance of aligning microbial inputs with soil nutrient status and community compatibility. The proposed model offers a theoretical basis for optimizing microbial interventions in plant–soil systems, where functional outcomes rely on microbial traits alongside their alignment with the local biogeochemical and ecological context. Although taxonomic composition was not directly assessed, the functional outcomes observed (particularly the high and stable metabolic diversity under csuftcsp75) suggest that microbial enhancement can occur without necessarily restructuring community composition. This supports a view of microbial inoculants not as displacers of native microbiota, but as ecological facilitators that integrate into existing networks, stimulate metabolic potential, and contribute to functional resilience.

## Figures and Tables

**Table 1:** Statistical methods used for each parameter.

Soil Property	Test Used
Total nitrogen	Kruskal–Wallis + Dunn
Total phosphorus	ANOVA + Tukey
Total potassium	Kruskal–Wallis + Dunn
Total carbon	Kruskal–Wallis + Dunn
Available nitrogen	Kruskal–Wallis + Dunn
Available phosphorus	ANOVA + Tukey
Available potassium	ANOVA + Tukey

## Data Availability

The original contributions presented in this study are included in the article/Supplementary Material. Further inquiries can be directed to the corresponding author.

## Supplementary Materials

The following supporting information can be downloaded at: https://www.mdpi.com/article/10.3390/microorganisms13071454/s1. Figure S1: Carbon Sources in EcoPlate. References [44,45,46,47,48,49,50,51,52,53,54] are cited in the Supplementary Materials.
