# Correction: Yang et al. Nutrient-Driven Metabolic Activation and Microbial Restructuring Induced by Endophytic *Bacillus* in Blight-Affected Forest Soils. *Microorganisms* 2025, *13*, 1454

**DOI:** 10.3390/microorganisms13112534

**Published:** 2025-11-05

**Authors:** Quan Yang, Shimeng Tan, Anqi Niu, Junang Liu, Guoying Zhou

**Affiliations:** 1 Key Laboratory of National Forestry and Grassland Administration on Control of Artificial Forest Diseases and Pests in South China, Central South University of Forestry and Technology, Changsha 410004, China; ywuqun@126.com (Q.Y.); kjc9620@163.com (J.L.); 2 Hunan Provincial Key Laboratory for Control of Forest Diseases and Pests, Central South University of Forestry and Technology, Changsha 410004, China; 3 Key Laboratory of Cultivation and Protection for Non-Wood Forest Trees, Central South University of Forestry and Technology, Changsha 410004, China; 4 State Key Laboratory of Utilization of Woody Oil Resource, Central South University of Forestry and Technology, Changsha 410004, China; 5 College of Forestry, Central South University of Forestry and Technology, Changsha 410004, China; 6 Key Laboratory of Plant Disease and Pest Control of Hainan Province, Institute of Plant Protection (Research Center of Quality Safety and Standards for Agricultural Products of Hainan Academy of Agricultural Sciences), Haikou 571100, China; tshmeng@126.com; 7 College of Life and Environmental Sciences, Central South University of Forestry and Technology, Changsha 410004, China

In the original publication [1], a declaration of the use of Generative AI was not included in the Materials and Methods section and the Acknowledgments section.

This information has been added to 2. Materials and Methods, 2.4. Use of Generative AI, Paragraph Number 1:

ChatGPT (version 4o) and Deepseek (version V3) were used for the purposes of language polishing, R code checking and summarizing the identified literature.

This information has been added to the Acknowledgments section:

During the preparation of this manuscript, the authors used ChatGPT (version 4o) and Deepseek (version V3) for the purposes of language polishing, R code checking and summarizing the identified literature. The authors have reviewed and edited the output and take full responsibility for the content of this publication.

The authors state that the scientific conclusions are unaffected. This correction was approved by the Academic Editor. The original publication has also been updated.
