# Supplementary material for: Nutrient-Driven Metabolic Activation and Microbial Restructuring Induced by Endophytic Bacillus in Blight-Affected Forest Soils
Source: Microorganisms. 2025 Jun 23;13(7):1454. doi: 10.3390/microorganisms13071454 (PMC12299972; doi:10.3390/microorganisms13071454)
Supplement: Supplementary file 1 [file microorganisms-13-01454-s001.zip › microorganisms-3652831-supplementary.pdf]

EcoPlate™

A1<br>Water	A2<br>β-Methyl-D-Glucoside	A3<br>D-Galactonic Acid γ-Lactone	A4<br>L-Arginine	A5<br>Water	A6<br>β-Methyl-D-Glucoside	A7<br>D-Galactonic Acid γ-Lactone	A8<br>L-Arginine	A9<br>Water	A10<br>β-Methyl-D-Glucoside	A11<br>D-Galactonic Acid γ-Lactone	A12<br>L-Arginine
B1<br>Pyruvic Acid Methyl Ester	B2<br>D-Xylose	B3<br>D-Galacturonic Acid	B4<br>L-Asparagine	B5<br>Pyruvic Acid Methyl Ester	B6<br>D-Xylose	B7<br>D-Galacturonic Acid	B8<br>L-Asparagine	B9<br>Pyruvic Acid Methyl Ester	B10<br>D-Xylose	B11<br>D-Galacturonic Acid	B12<br>L-Asparagine
C1<br>Tween 40	C2<br>i-Erythritol	C3<br>2-Hydroxy Benzoic Acid	C4<br>L-Phenylalanine	C5<br>Tween 40	C6<br>i-Erythritol	C7<br>2-Hydroxy Benzoic Acid	C8<br>L-Phenylalanine	C9<br>Tween 40	C10<br>i-Erythritol	C11<br>2-Hydroxy Benzoic Acid	C12<br>L-Phenylalanine
D1<br>Tween 80	D2<br>D-Mannitol	D3<br>4-Hydroxy Benzoic Acid	D4<br>L-Serine	D5<br>Tween 80	D6<br>D-Mannitol	D7<br>4-Hydroxy Benzoic Acid	D4<br>L-Serine	D9<br>Tween 80	D10<br>D-Mannitol	D11<br>4-Hydroxy Benzoic Acid	D12<br>L-Serine
E1<br>α-Cyclodextrin	E2<br>N-Acetyl-D-Glucosamine	E3<br>γ-Amino Butyric Acid	E4<br>L-Threonine	E5<br>α-Cyclodextrin	E6<br>N-Acetyl-D-Glucosamine	E7<br>γ-Amino Butyric Acid	E8<br>L-Threonine	E9<br>α-Cyclodextrin	E10<br>N-Acetyl-D-Glucosamine	E11<br>γ-Amino Butyric Acid	E12<br>L-Threonine
F1<br>Glycogen	F2<br>D-Glucosaminic Acid	F3<br>Itaconic Acid	F4<br>β-Hydroxy-Glycyl-L-Glutamic Acid	F5<br>Glycogen	F6<br>D-Glucosaminic Acid	F7<br>Itaconic Acid	F8<br>β-Hydroxy-Glycyl-L-Glutamic Acid	F9<br>Glycogen	F10<br>D-Glucosaminic Acid	F11<br>Itaconic Acid	F12<br>β-Hydroxy-Glycyl-L-Glutamic Acid
G1<br>D-Cellobiose	G2<br>Glucose-1-Phosphate	G3<br>α-Keto Butyric Acid	G4<br>Phenylethyl-amine	G5<br>D-Cellobiose	G6<br>Glucose-1-Phosphate	G7<br>α-Keto Butyric Acid	G8<br>Phenylethyl-amine	G9<br>D-Cellobiose	G10<br>Glucose-1-Phosphate	G11<br>α-Keto Butyric Acid	G12<br>Phenylethyl-amine
H1<br>α-D-Lactose	H2<br>D,L-α-Glycerol Phosphate	H3<br>D-Malic Acid	H4<br>Putrescine	H5<br>α-D-Lactose	H6<br>D,L-α-Glycerol Phosphate	H7<br>D-Malic Acid	H8<br>Putrescine	H9<br>α-D-Lactose	H10<br>D,L-α-Glycerol Phosphate	H11<br>D-Malic Acid	H12<br>Putrescine

Figure S1. Carbon Sources in EcoPlate

INTRODUCTION

Microbial communities provide useful information about environmental change. Microorganisms are present in virtually all environments and are typically the first organisms to react to chemical and physical changes in the environment. Because they are near the bottom of the food chain, changes in microbial communities are often a precursor to changes in the health and viability of the environment as a whole.

The Biolog EcoPlate™ (Figure S1) was created specifically for community analysis and microbial ecological studies. It was originally designed at the request of a group of microbial ecologists that wanted a panel that provided replicate sets of tests[1].

Community analysis using Biolog microplates was originally described in 1991 by J. Garland and A. Mills[2]. They and other researchers found that by inoculating a mixed population of microorganisms and measuring the community metabolism over time, they could ascertain characteristics of that community. This approach, called community–level physiological profiling, or CLPP, has been demonstrated to be effective at distinguishing spatial and temporal changes in microbial communities. In applied ecological research EcoPlates are used as both an assay of the stability of a normal population and to detect and assess changes following the onset of an environmental variable.

Studies have been done in diverse applications of microbial ecology and have demonstrated the fundamental utility of EcoPlates in detecting population changes in soil, water, wastewater, activated sludge, compost, and industrial waste. The utility of the information has been documented in hundreds of publications using Biolog technology to analyze microbial communities. A bibliography of publications is posted on the Biolog website at www.biolog.com/support/publication-database.

ECOPLATE

The EcoPlate contains 31 carbon sources that are useful for community analysis. These 31 carbon sources are repeated 3 times to provide more data replicates. Communities of microorganisms will give a characteristic reaction pattern called a metabolic fingerprint. From a single EcoPlate, these fingerprint reaction patterns rapidly and easily characterize the community.

The community reaction patterns are typically analyzed at defined time intervals over 2 to 5 days. The changes in the pattern are compared and analyzed using statistical analysis software. The most popular method of analysis of the data is via Principle Components Analysis (PCA) of average well color development (AWCD) data, but alternative methods may also offer advantages[3-11]. The changes observed in the fingerprint pattern provide useful data about the microbial population changes over time.

TYPICAL PROCEDURE[3]

STEP 1: Environmental samples are inoculated directly into EcoPlates either as aqueous samples or after suspension (soil, sludge, sediment, etc.).

STEP 2: The EcoPlates are incubated and kinetic data collected and analyzed.

STEP 3: The community-level physiological profile is assessed for key characteristics:

- Pattern development (similarity)
- Rate of color change in each well (activity)
- Richness of well response (diversity)

Formation of purple color occurs when the microbes can utilize the carbon source and begin to respire. The respiration of the cells in the community reduces a tetrazolium dye that is included with the carbon source.

The reaction patterns are most effectively analyzed using an Odin™ system for Phenotype Characterization. Odin can incubate and read up to 50 plates at once, and automatically generates kinetic analyses for each condition.

Statistical analysis of the data is typically performed using standard software packages. Some researchers have found that PCA provides greater resolution than other methods of statistical analysis[11].

EcoPlates: Catalog No. 1506 (10/box)

REFERENCES

[1] A new set of substrates proposed for community characterization in environmental samples. H. Insam, p. 260-261, In: Microbial Communities. Functional versus structural approaches, H. Insam and A. Rangger, editors, 1997, Springer.

[2] Classification and characterization of heterotrophic microbial communities on the basis of patterns of community level sole-carbon-source utilization. J.L. Garland, A.L. Mills, Applied and Environmental Microbiology, 1991, v.57, p. 2351-2359.

[3] Analysis and interpretation of community-level physiological profiles in microbial ecology. J.L. Garland, Federation of European Microbiological Societies, Microbiology Ecology, 1997, v. 24, p289-300.

[4] Community analysis by Biolog: curve integration for statistical analysis of activated sludge microbial habitats, J.B. Guckert, G.J. Carr, T.D. Johnson, B.G. Hamm, D.H. Davidson, Y. Kumagai, Journal of Microbiological Methods, 1996, v. 27:2-3, p. 183-187.

[5] Statistical analysis of the time-course of Biolog substrate utilization. C.A. Hackett, B.S. Griffiths, Journal of Microbiological Methods, 1997, v. 30, p. 63-69.

[6] Statistical comparisons of community catabolic profiles. E. Glimm, H. Heuer, B. Engelen, K. Smalla, H. Backhaus, Journal of Microbiological Methods, 1997, v. 30, p. 71-80.

[7] Application of multivariate analysis of variance and related techniques in soil studies with substrate utilization tests, W. Hitzl, M. Henrich, M. Kessel, and H. Insam, Journal of Microbiological Methods, 1997, v. 30, p. 81-89.

[8] Using the Gini coefficient with BIOLOG substrate utilization data to provide an alternative quantitative measure for comparing bacterial soil communities, B.D. Harch, R.L. Correll, W. Meech, C.A. Kirkby, and C.E. Pankhurst, Journal of Microbiological Methods, 1997, v. 30, p. 91-101.

[9] Monitoring soil bacteria with community-level physiological profiles using Biolog EcoPlates in the Netherlands and Europe, Michiel Rutgers, Marja

Christian Mulder, Dorothy Stone, Rachel E. Creamer, Anne Winding and Jaap Bloem, Applied Soil Ecology, 2016, v. 97, p. 23-35.

[10] Community-level physiological profiling. K.P. Weber and R. L. Legge, p. 263-281, In: Bioremediation, Methods in Microbial Ecology v. 599, S.P. Cummings, editor, 2010, Springer.

[11] Defining soil quality in terms of microbial structure. M. Firestone, T. Balser, D. Herman, Annual Reports of Research Projects, UC Berkeley, 1997.

biolog

21124 Cabot Blvd.
Hayward, CA 94545

biolog.com
+1 800 284 4949

EcoPlate and Odin are trademarks of Biolog, Inc., Hayward, CA
